# Framing a Knowledge Base for a Legal Expert System Dealing with Indeterminate Concepts

**DOI:** 10.1155/2015/985425

**Published:** 2015-10-01

**Authors:** Michał Araszkiewicz, Agata Łopatkiewicz, Adam Zienkiewicz, Tomasz Zurek

**Affiliations:** ^1^Faculty of Law and Administration, Jagiellonian University, Gołębia 24, 31-007 Kraków, Poland; ^2^Faculty of Philosophy, Jagiellonian University, Ulica Gołębia 24, 31-007 Kraków, Poland; ^3^Faculty of Law and Administration, University of Warmia and Mazury, Ulica Warszawska 98, 10-702 Olsztyn, Poland; ^4^Institute of Computer Science, Maria Curie-Sklodowska University, Ulica Akademicka 9, 20-033 Lublin, Poland

## Abstract

Despite decades of development of formal tools for modelling legal knowledge and reasoning, the creation of a fully fledged legal decision support system remains challenging. Among those challenges, such system requires an enormous amount of commonsense knowledge to derive legal expertise. This paper describes the development of a negotiation decision support system (the Parenting Plan Support System or PPSS) to support parents in drafting an agreement (the *parenting plan*) for the exercise of parental custody of minor children after a divorce is granted. The main objective here is to discuss problems of framing an intuitively appealing and computationally efficient knowledge base that can adequately represent the indeterminate legal concept of the well-being of the child in the context of continental legal culture and of Polish law in particular. In addition to commonsense reasoning, interpretation of such a concept demands both legal expertise and significant professional knowledge from other domains.

## 1. Introduction

From their inception, expert systems have been seen as a suitable tool for decision support in legal contexts [1, 2]. However, while the first attempts to create such systems seemed promising [3], deeper inquiry revealed a number of difficulties [4]. While it is not our concern here to elaborate the problems associated with the representation of legal knowledge in computer systems, it is our opinion that the most difficult task in that context is to model the commonsense knowledge and contextual reasoning mechanisms widely used by legal practitioners. Without such elements, expert systems could only be used to support decisions in the most trivial cases. A few important aspects of the modelling of commonsense knowledge in legal decision support systems are worthy of further discussion here: the modelling of indeterminate concepts widely used in legal practice and the representation of commonsense rules reflecting the mode of reasoning used by human lawyers. It is trivial to observe that commonsense rules differ from strict, logic-based rules in that they are, for instance, usually defeasible, uncertain, and sometimes conflicting. The nature of such rules and the relations between them has long been a topic of debate ([5, 6] and many others).

Among many and varied previous conceptions of legal decision support tools ([3, 7] and others), we will focus here on one specific approach in developing a tool to support negotiation between parents in drafting a plan for parental custody arrangements for minor children following a divorce. In particular, the present paper describes our attempt to formalise currently informal mechanisms for evaluation of a child's well-being, which requires some discussion of salient aspects of the formalisation of legal and commonsense knowledge. Appropriate utilisation of various forms of negation as well as determination of orders between rules is crucial for the accuracy of models of knowledge. The main objective here is to discuss how an intuitively appealing and computationally efficient knowledge base can be framed to deal with such indeterminate legal concepts and how the preference relations between rules used to interpret this concept in concrete cases can be determined.

The present investigation is structured as follows.  Section 2 outlines the structure of the Parenting Plan Support System (PPSS).  Section 3 compares PPSS with existing online dispute resolution systems (mainly those dealing with issues of family law) and details the research problem here.  Section 4 considers the possibility of generalising features of individual cases from a practical legal perspective, referring mainly to civil law culture, where, in principle, there are no binding precedents or derived argumentative schemes. In Section 5, the notion of heuristics as understood in cognitive science is discussed, as well as the kinds of move used in the AI and law literature to establish preference relations between the elements of relevant knowledge bases.  Section 6 presents a technical discussion of the syntax of defeasible rules—that is, the possibility of utilisation of negation in their antecedents—and discusses the notion of preference between rules. Finally, Section 7 presents our conclusions.

## 2. The Parenting Plan Support System

PPSS [8–10], partially implemented in [11], is a negotiation decision support system to support parents in drafting an agreement (the* parenting plan*) to govern their exercise of parental custody over minor children after a divorce is granted under Polish law.

### 2.1. The Parenting Plan: Legal and Conceptual Context

In a divorce judgment, the Polish family court determines parental authority over a minor child and parties' contact with the child, and issues regarding the financial costs of maintenance and raising the child are decided. The court recognises such an agreement between the spouses only if it is consistent with the “well-being of the child.” The court may grant parental authority to only one of the parents, confining the other parent to specified duties and rights in respect of the child. Alternatively, provided they have submitted a parenting plan, the court may grant parental authority to both parents, on the assumption that they will cooperate on matters regarding the child (art. 58 §1 and 1a of the Polish Family and Custody Code, hereafter PFCC). A similar regulation is applicable in the case of unmarried parents living separately (art. 107 PFCC). In abstract terms, Polish law does not favour any specific allocation of parental authority; the issue is always decided on the concrete circumstances of a case. In this context, it is important to note the constitutional and statutory principle of the autonomy of parental authority in Polish law (art. 48 par. 1, art. 53 par. 3, and art. 70 par. 3 of the Polish Constitution and art. 97 §1 PFCC).

The fundamental issues of relevance to the parenting plan as agreed by the parents (sometimes with participation of a third person such as a mediator) are parental authority, contact between parents and child, and the child's well-being. These in turn relate to “background” concepts such as public interest, the interests of the parents, and the rational cooperation of parents and child. According to Polish law, parental authority is to be defined as the duty and right of the parents to have custody over the person and property of the child, with due regard to the child's dignity and rights. Children subordinate to parental authority owe obedience to their parents, and even where the children are competent enough to act independently, they should take into account their parents' advice, provided that this advice is formulated for the sake of the child's well-being. Parental authority should be exercised in accordance with the child's well-being and with public well-being, and any reasonable wishes of the child should be taken into account under certain conditions (art. 95 PFCC). The parents must raise and direct the child under their parental authority, taking care of the child's physical and spiritual development and preparing him or her to work as a member of society in accordance with his or her abilities. The child remains under parental authority until reaching legal maturity, typically at 18 years of age. In principle, parental authority is vested in both parents (art 92 and 93 PFCC). Independent of parental authority, both parents and child have the right and duty to remain in contact. This contact with the child encompasses, inter alia, spending time with the child, direct communication, and distance communication (e.g., by means of electronic devices) (art 113 PFCC).

Parental authority should be exercised in accordance with the child's well-being, in the public interest (95 §3 PFCC) and with respect to the dignity and rights of the child as specified in the UN Convention on the Rights of the Child (adopted on 20 November 1989). In consequence, the child's well-being should be considered central and prerequisite in the exercise of parental authority and contact between the parties. This is determined in light of the concrete circumstances of a given case to ensure the proper personal (spiritual) development of the child (Order of the Polish Supreme Court 11 January 2000, I CKN 327/98). The concept of the child's well-being relates to the concept of the prevailing interest of the child, construed on the basis of the UN Convention on the Rights of the Child (Preamble to the Convention).

The second major criterion is the public interest. The child's upbringing is also a realisation of the parents' social function, taking care of the child's physical and spiritual development to prepare him or her to work for the benefit of the society, according to his or her capacities (art. 96 §1 PFCC). The Convention also takes into account these matters, albeit with important caveats (cf. the Convention and the Resolution of the PSC of 12 June 1992, III CZP 48/92).

As a consequence, in deciding on the issue of parental authority, the court should be guided first and foremost by the child's well-being (with due regard to the public interest) rather than solely or mainly by the interests of one or both parents (cf. Resolution of the PSC of 12 June 1992, III CZP 48/92, and Judgment of the PSC of 25 August 1981, III CRN 155/81). The interests of the parents remain important but cannot be decisive; the court should, if possible, take into account the position and interests of the parents (Order of the PSC of 5 May 2000, II CKN 765/00, and Resolution of the PSC of 09 June 1976, III CZP 46/75). This approach is consistent with the Convention on the Rights of the Child (cf. Resolution of the PSC of 12 June 1992, III CZP 48/92, and Resolution of the PSC of 08 March 2006, III CZP 98/05). The principle of rational cooperation between parents and child must also inform the exercise of parental authority (cf. art. 72 par. 3 of the Polish Constitution, art. 95 §2 and 4 of the PFCC).

Summarising the legal and conceptual context in which the parenting plan is developed, the following points should be noted:

1. If the divorcing parents (or unmarried parents living apart) intend that parental authority be vested in both of them, they should prepare an agreement (the parenting plan) governing their exercise of parental authority and contact with the child.
2. The plan is assessed by the court, principally on the criterion of the child's well-being and with reasonable expectations of parental cooperation with regard to the child. However, the court must also take into account other criteria, including the public interest and the interests of the parents.
3. The plan is in principle adopted as a particular type of agreement rather than as a formal settlement and does not entail any substantive legal results. Instead, the plan is no more than a premise to be taken into account by the court in order to vest parental authority in both of the parents. The court alone is authorised to decide on the rules governing the exercise of parental authority, and the plan is not binding on the court.
4. The plan is approved by means of a judgment granting parental authority to both parents. In cases where the plan is not accepted the court decides on matters of parental authority, based on the totality of the case's circumstances and taking into account any proposals submitted by the parents in the plan.
5. Polish law does not prescribe any particular form or content for the parenting plan.

The legal context for the decision support system described in this paper involves rules of law that prescribe no ready answers to issues arising in drafting a parenting plan. Presumably, no uniquely “right” plan exists in any concrete situation, and this legal domain can certainly be characterised as ill-defined [12]. The system described here aims to revisit the domain to provide interested parties with a tool that may be of use in resolving problems that arise in drafting the parenting plan.

### 2.2. The Parenting Plan Support System: Functional and Structural Context

In preparing a parenting plan, the task for parents is to devise an agreement that can satisfy judicial assessment of whether it ensures the child's well-being (as interpreted in the concrete circumstances) and of whether it can reasonably be expected to be realised. More specifically, the functions of such a plan are as follows:

- To facilitate regulation of relations between parents and child following divorce.
- To secure the child's interests.
- To eliminate any unacceptable behaviour of the parents.
- To educate the parents with regard to pedagogical, legal, psychological, and social principles informing their relations with the child.
- To prevent future judicial disputes concerning the child's well-being.

It is worth noting that this task is made more difficult because the parents are in conflict and are not usually professionally equipped to draft a parenting plan.

In determining the content of a parenting plan, the argumentative discourse exhibits four main dimensions: communicational, informational, relational, and decisional. The preferred negotiation strategy is nonadversarial and cooperative, characterised by a win-win paradigm as against an adversarial, win-lose competitive approach. The final version of PPSS should provide the following support for parents in performing these tasks:

1. Providing the parties with a template of sufficient complexity for the parenting plan, adjustable to the concrete circumstances of a case and with a simple tutoring module that sets out the functions and aims of the system.
2. Enabling users to input salient data concerning the child and relations between parents and child, as well as other relevant data.
3. Including information about the nature of any conflict between the parents and, in particular, whether that conflict relates to the parents' respective values.
4. Providing the parents with a rich variety of (contextually relevant) options to clarify the scope and limits of their choice.
5. Encouraging the parents to adopt a cooperative attitude by providing suggestions concerning their choices and possible mutual concessions.
6. Enabling the negotiating parents to communicate, allowing them to deliberate directly on some choices while opting for automated generation of other parts of the plan.
7. Establishing the space of agreement concerning contextual data provided by the parents, enabling negotiation of options that are mutually acceptable and mutually advantageous and realistic.
8. Grounding any suggestions provided by the system (and ultimately by the negotiated parenting plan) in an interdisciplinary knowledge base encompassing psychological, pedagogical, and legal data.
9. Assessing any options chosen by the parents in terms of the child's well-being, in a way that predicts the likely judicial assessment of the plan.
10. Generating a final electronic version of the parenting plan to be submitted to the court. The system should also be able to update the plan where future circumstances change.

This paper is concerned with the system's conceptual architecture rather than its technological aspects. Although the system has not as yet been fully implemented, some initial attempts have been made [11]. An important subset of the system's knowledge base has been extracted from thirty actual and influential judgments by the Polish Supreme Court, yielding a large number of salient factors that will play a crucial role in the operation of the system, as detailed below. A case analysis based on an existing (unofficial) sample form of the parenting plan made it possible to systematise the content of the parenting plan into ten parts, referred to as “categories.” Additional special categories—relating, for instance, to any incapacities of the child—are omitted here. For each parenting plan, data referred to as* Essential Information* must be entered. For the sake of simplicity, we assume here that the parents have only one child. On that basis, the structure of the parenting plan is as follows.

Essential information:

- Formal (data relating to the parties, important dates, etc.),
- emotional (relating to emotional relations between the actors involved),
- implementation (declarations relating to realisation of the agreement after its execution).

The specific parts of the plan are structured as follows (more detailed discussion is presented in [8]):

 Category 1: contact between parents and the child.
 Category 2: contact between the child, family members, and other people.
 Category 3: education.
 Category 4: livelihood.
 Category 5: leisure, holidays, and ceremonies.
 Category 6: worldview and childrearing rules.
 Category 7: child health.
 Category 8: information and documentation.
 Category 9: proceeding in emergency cases.
 Category 10: any other issues.

The list of categories (i.e., issues to be included in the parenting plan for completeness) is based on informal templates for such agreements that are used in actual practice. The structure of the agreement is mirrored in the knowledge representation structures of PPSS.

### 2.3. Structure of PPSS

PPSS consists principally of the following modules:

- The Questionnaire asks users to provide basic information about their situation (e.g., age and number of children, income, and familial relationships). The system suggests answers, but no NLP support is offered in the present version. As a result, data relating to the so-called* Environmental Factors* are stored in the system. These propositional elements are essential to contextualise the assessment of options chosen by users in the next module.
- The Option Choice Module presents proposals to users relating to contractual clauses to be incorporated in the parenting plan. Most importantly, the system assesses any options chosen by users in terms of the well-being of the child. Such assessment is made possible by the rule-based inference engine, which takes as input the Environmental Factors introduced by users and the options they choose, generating an evaluation of these options as output. This part of the system has been partially implemented [11]. The development of this module is of central interest here.
- The Option Deliberation Module enables users to compare the options they have chosen and to enter into a bargaining phase. If users can agree on a set of contractual provisions, PPSS generates an agreement (i.e., a parenting plan) as an output. This part of PPSS exists only as a theoretical model (see [8, 9] for a detailed description), and work is in progress on its implementation. The functioning of this module is not central to the present contribution.
- The Explanation Module is a standard component of expert systems, providing users with reasons for system inferences and outputs.

One important element of the Option Choice Module is a rule-based expert system for interpretation of the concept of well-being of the child. This part of the system is of utmost importance because, under Polish law, the parenting plan can be accepted by the court only if that plan accords with the criterion of well-being of the child. One of the main functions of the system is to indicate to users whether the provisions of their chosen agreement are in accordance with this criterion, which remain an indeterminate concept. The relevant literature identifies several types of indeterminateness of legal concepts [13]:* open-textured* (where the emergence of new situations in the world may change its initial boundaries);* contextual* (where the meaning and scope of the concept change with context); and* evaluative* (where investigations concerning meaning and scope of legal concepts meaning and scope depend significantly on the individual evaluations of the agents involved). This indeterminateness raises the question of how a concept of this type can be modelled in an expert system. One obvious requirement is that the system's knowledge base should include a set of exemplary cases, explaining both prototypical and borderline instances of the concept's application. This is perfectly embodied in the Case-Based Reasoning (CBR) approach in the AI and law literature, which emerged in the USA in the 1980s and has continued to evolve [14–16], leading to the development of hybrid systems that combine rule-based and case-based elements [17]. Another important line of development relates to the introduction of elements of teleological reasoning into the knowledge base [18–20]. Although fruitful, these ideas are not directly applicable to legal expert systems rooted in continental legal culture (as opposed to common law culture), where there is no doctrine of binding precedent, and judges—who often take a formalistic approach—are not inclined to engage in discussion of the values underlying their decisions.

The PPSS knowledge base (presented in detail in [9]) encompasses, in particular, the following elements.

*Options*. These include any propositions that may form part of the parenting plan. Options are grouped under Questions, each of which provides for a set of options. The assumption is that, in any developed agreement, one and only one Option from each Question should be chosen.

*Dimensions*. PPSS contains a library of ten dimensions, each related to one of the important broad issues (categories) that inform the content of any parenting plan. More detailed lists of the dimensions can be found in [9, 11]. Each dimension comprises a ten-step ordinal scale [0–9] (from least favourable to most acceptable with respect to the well-being of the child).

*Environmental Factors (EFs)*. This set encompasses sentences containing basic information about people involved in the dispute, as well as a basic description of the case. Users generate a concrete set of EFs by making choices in the Questionnaire module of PPSS.

*Defeasible Rules (DRs)*. As a generalised account of DRs (modified with respect to the earlier view presented in [9, 11]), let DR(*Di*) be a set of defeasible rules assigned to a Dimension *i*. A rule belongs to this set if and only if it has the following form:

(1) $$\begin{array}{c} \Omega ⟹\left[\;0\leq n\leq 9\;\right]\left(\;Di\;\right), \end{array}$$

where

(i) *Ω* is a finite conjunctive formula encompassing elements from the set of options and EFs such that the set of options includes at least one element (the set of EFs may be empty);
(ii) [0 ≤ *n* ≤ 9](*Di*) is the valuation of the antecedent of the DR in question with regard to one of the dimensions of PPSS.

Defeasible rules are not rules in the traditional meaning of this word (see, e.g., [6, 21]), nor are they (valuation) functions because of their defeasibility. They are conditionals, in which satisfaction of antecedents does not entail truthfulness of a conclusion but establishes the level of estimation of a given dimension. In other words, DRs adopt conjunctions of options and EFs as their input and generate a value from the ten-step ordinal scale as their output. It follows that the set of defeasible rules within PPSS may be defined as a set of relations adopting elements from the power set of the set of all propositional elements (options and EFs) in the knowledge base (PROP) as its domain and the elements of the set of values (*V*) encompassing the ten elements (from 0 to 9) as its codomain:

(2) $$\begin{array}{c} \text{DR}=R\langle \;p\in P\left(\;\text{PROP}\;\right),v\in V\;\rangle . \end{array}$$

Additionally, we define a preference relation for the set of rules (see Section 6 for a formal exposition). If two rules, *r*_1_ and *r*_2_, belong to the relation of preference, and *r*_1_ is preferred over *r*_2_, then, where both are applicable to a given factual situation, the consequent of *r*_1_ is derived and the consequent of *r*_2_ should be rejected. On that basis, the evaluation of a given option can be performed in the following way.

For every option, a set of defeasible rules should be chosen whose conditions are satisfied by a given case. If such set is empty, the evaluation of that option cannot be performed; if there is one rule, that rule becomes the preferred one; if there is more than one rule, the preferred one should be chosen on the basis of the order ORD. If preferred rules are chosen for all options, the option attracting the highest level of evaluation for a given dimension is optimal. Where users opt for other solutions, they are informed of the suboptimal character of their choice.

Forming the most important part of the Option Choice Module, the inference engine operating on a knowledge base generates assessments for users about the options they have chosen in terms of its value for the well-being of the child. As understood by the Polish judiciary and in the literature on pedagogy and developmental psychology, this is considered paramount. As explained in [10], users may react in different ways to valuations provided by the system's inference engine; that is, they may agree or disagree with the information provided by PPSS. In cases of disagreement with the system's suggestions, users may be asked to provide grounds for their position by choosing one of the possible answers suggested by the system. If the information provided by PPSS is assessed as irrelevant by users (e.g., because the contextual information stored in the knowledge base is insufficient to grasp the actualities of the case), they may engage in communication with each other by means of a dialogue panel. The information provided by this module is important for the process of negotiation because it informs the parties about available alternatives to the negotiated agreement.

Once sets of options have been chosen, parents may move to the next module of the system: the Option Deliberation Module (ODM). Described in [8, 9], this module has not yet been implemented but exists as a theoretical model. The ODM enables users to check whether the options they have chosen are within their space of agreement and to enter into a bargaining phase. The system enables both manual and semiautomated allocation of options, and users are asked to assign numerals to the disputed options (the sum of the points assigned by each user is normalised to 100). PPSS favors those allocations that maximise the equality of distribution of preferred options among users, once they do not violate the value of well-being of the child. The eventual output of the system is a proposal characterised by complete parental agreement.

## 3. Comparison of PPSS to Related Work and Formulation of Research Problem

As outlined in the preceding section, PPSS is an online dispute resolution (ODR) model that integrates three artificial intelligence techniques: NDSS (Negotiation Decision Support Systems), RbS (Rule-based Systems), and CBR (Case-Based Reasoning) (see [22] for a general discussion of the application of AI techniques in ODR systems). With regard to the latter two components, PPSS acts as a classic expert system, providing answers for users of the system concerning the acceptability of their chosen parenting plan provisions in terms of the indeterminate criterion of the child's well-being. In order to define the scope of the contribution of the PPSS project, it is necessary to compare it to existing work in the field.

The first important comparison is provided by work in the field of Negotiation Support Systems. In the domain of family law, the systems developed by Zeleznikow and his collaborators, such as Family_Winner [23], Asset Divider [24], and IMODRE [25], came to prominence in the relevant literature. The PPSS Option Deliberation Module (still a theoretical model) performs functions similar to those of the well-known Family_Winner program. This latter system is well known, assisting mediators and parties in family disputes concerning the allocation of certain important items, both monetary and nonmonetary (such as custody of the child). Family_Winner uses game theory and heuristics to generate tradeoff maps, representing a party's preferences and tradeoffs. Items to be divided are entered by the users themselves; the values that are taken into account in the process of negotiations reflect the interests and preferences of the negotiating parties. As noted by Abrahams et al. [25], this facet of Family_Winner has been discussed with representatives of Victoria Legal Aid in Australia, and it was suggested that the authors should incorporate the notion of fairness in their further work, reflecting the paramount interest of children. Inter alia, this led to the creation of the Asset Divider program and a full-fledged multiagent system called IMODRE, in which an Asset Divider-based agent serves as one among many cooperating artificial agents.

It is clear that the line of development of such systems proceeds from the classic Negotiation Support Systems paradigm to incorporate elements of legal knowledge. The point of departure for PPSS was exactly the opposite, structuring the legal knowledge base relating to interpretation of the concept of the well-being of children (similar to the paramount interest of children in Australian family law) and leaving the implementation of the NDSS module of the system for future research. PPSS does not generally allow users to name issues or items negotiated; instead, it requires parties to reflect on all the important clauses of the potential parental agreement. Such a methodological choice has serious consequences; it is noted in the literature that Family_Winner and Asset Divider are, to a large extent, domain-independent [23, 26] while PPSS is obviously strictly domain-dependent, as Environmental Factors, options, and defeasible rules will be inapplicable to other domains of negotiation.

Given that the current work on PPSS focuses on the RbS and CBR components rather than on the NSS, more detailed comparison with existing solutions is not possible. It is worth noting, however, that PPSS embodies the desiderata for ODR environments as formulated by Lodder and Zeleznikow [27], who suggest that such systems should be able to perform the following functions:

- Calculating best alternatives to negotiated agreements (BATNAs) for the parties.
- Enabling the parties to communicate with each other to discuss disputed issues in a process of dialogue.
- Proposing how disputed items are to be allocated among the parties.

Calculation of BATNAs and of other types of alternatives to negotiated agreements such as WATNAs (worst alternatives to negotiated agreement) and MLATNAs (most likely alternatives to negotiated agreement) is among the most important topics in the development of NSS and ODR tools in general (see, e.g., [28]). PPSS, which offers users an assessment of their chosen options in relation to the legal criterion of well-being of the child, advises divorcing parents of how their preferred clauses are likely to be assessed by the court and of the clauses that would be probably favoured by the court. The presence of certain environmental factors may also play a role in predicting judicial rulings in cases of lack of agreement between spouses; for instance, where there has been a history of violence towards the child by one of the users, it is highly likely that their parental authority will be limited or even refused.

As indicated, PPSS enables users to communicate with each other. However, this is not the most important feature of the system, as it focuses on a legal assessment of parents' preferred options more than on satisfaction of those preferences. As noted earlier, PPSS also provides a module for allocation of the discussed items (options). While the desiderata formulated by Lodder and Zeleznikow are met, PPSS places strong emphasis on legal assessment of the deliberated agreement rather than on mechanisms to support realisation of parties' interests.

The output generated by PPSS might be interpreted as advice for the involved parties or mediators. In this respect, it may be compared to Split-Up (see, e.g., [29]), a well-known decision support system used in family law cases involving the division of marital property. An important difference between PPSS and Split-Up is that while the latter is a hybrid system, combining rule-based mechanisms and connectionist networks, PPSS exclusively uses traditional symbolic propositional representations. This choice is dictated by the educational function of PPSS, requiring that all steps of inference performed by the program should be explicable to the users to persuade them to follow partial items of advice, leading eventually to the final output. Such functions cannot be performed by subsymbolic connectionist representations, which can however perform very fine-grained inferences, thus providing adequate advice or accurate prediction. Here, however, the aim is to explore the potential of classical hybrid systems rather than the application of other technologies to the problem.

On that basis, the most important comparators for the PPSS project are the classical CBR and hybrid systems that use representations based on dimensions and/or factors [14, 16, 17, 30, 31], as well as recent developments in the field of ODR that make extensive use of CBR inference mechanisms [32]. It should first be emphasised that the account of dimensions employed in PPSS differs significantly from the classical account presented in the HYPO system [16]. Briefly, HYPO dimensions are complex knowledge representation structures used to index cases and build arguments for or against the disputant's interest. These dimensions range from extremely proplaintiff to extremely prodefendant. They enable the system to retrieve cases similar to the case at issue (with respect to the set of shared dimensions) and to compare those cases in order to build analogy-based and distinguishing arguments, as well as counterexamples. On the other hand, CATO-style factors may or may not be present in a given case; if present, they support the position of a given party. Both approaches have their advantages and disadvantages, which cannot be discussed in depth here (but see [33]). PPSS combines these approaches, using both unary factors (Environmental Factors have this characteristic) and dimensions. The differences between HYPO and PPSS dimensions are as follows. Because PPSS assigns one dimension to each category of the parenting plan, each agreement will be indexed by every dimension stored in the system. PPSS dimensions encompass sets of propositional elements (Environmental Factors and options) with a certain valuation on the given dimension. Because each PPSS dimension involves a ten-step scale, it may be defined as a set of ten sets of propositional elements; sets belonging to a given step of the scale have the same valuation. The construction and application of HYPO dimensions are different because their role is to enable analogy-based argumentation. In typical situations, then, only a few dimensions will be present in a given case. HYPO dimensions also involve different scales (binary, ordinal, etc.).

Most importantly, knowledge structures in the present version of PPSS do not enable a fully fledged CBR, which would encompass retrieval of cases, analogizing from one case to another and forming arguments based on similarities and differences. Instead, these structures serve to enhance the contextualised character (Environmental Factors) and systematisation (dimensions) of rule-based reasoning, performed by means of defeasible rules in the assessment of particular clauses of the parenting plan. The use of CBR structures in PPSS differs considerably, then, from their use in existing ODR projects such as UMCourt [32], in which the information that constitutes a case encompasses the following categories:

- Problem—encompassing the following information types: background (similar to PPSS Environmental Factors), objectives (aims of the parties), and legal (in particular, applicable norms) and important dates in the case.
- Solutions—list of actions performed by the parties in order to achieve the outcome.
- Outcome—list of items describing the outcome in terms of indemnities to be paid and a value denoting the percentage of successful applications of this case to the dispute resolution process.

UMCourt uses different types of similarity metrics for retrieval of cases, including nearest neighbour and cosine similarity. As an output, the user obtains a list of potentially applicable cases, ordered in terms of their similarity. In particular, if two cases are decided on the basis of the same legal norm, those cases will be considered similar; elements determining the similarity of legal cases may have different weights.

Although this approach to the use of CBR in ODR systems such as UMCourt is valuable, it has limited application to the PPSS project because of the latter's peculiarities. Specifically, the role of PPSS is not to provide the parties with material for arguments in favour of one or other side of the dispute but to enable parents to draft a parenting plan which would optimise realisation of the child's well-being. It follows that results of stored cases (in terms of winning or losing party) would not be useful for the purposes of PPSS. Additionally, proximity of dates will not always indicate cases' similarity, as an old case may well contain an adequate and applicable interpretation of the child's well-being. In respect of preparation of the parenting plan, the governing legal norm will be derived from art. 58 §1 and 1a of the PFCC, as referred to in Section 2, which means that retrieval of cases on the basis of similar legal grounds would be trivial for PPSS.

In order to enhance PPSS with actual CBR tools, enabling retrieval, comparison, and argument from cases, a different approach is needed, in which the actual cases stored in the database should contain as much information as possible about interpretation of the concept of the child's well-being in the circumstances of the case. A first approach to this problem may be found in the work of Araszkiewicz et al. [11], on the basis of which the following questions can be asked:

1. How should the relevant information for interpretation of the child's well-being in a given case be represented in the knowledge base of the system?
2. How might this information be applied to other cases, given the limited applicability of both classic factor-based and dimension-based approaches and recent findings concerning the use of CBR in ODR systems?

Recall that the practical idea behind the knowledge structure in the Option Choice Module of PPSS is that parents interested in testing a certain option can obtain a valuation of that option in terms of the child's well-being, based on Environmental Factors they have introduced. This raises the question of how the set DR, as potentially applicable to a given case, should be represented within the system. An exhaustive approach would involve determining a finite set of all propositional elements before deciding on the valuation of each element of the set *P*(PROP). However, this would be unfounded in terms of descriptive adequacy, as it would be impossible to identify authoritative sources (judgments or scientific works) to justify valuations for all the possible inputs. For instance, if the set PROP encompassed 10 elements (a simple case), it would be necessary to determine the valuation of 1024 (i.e., 2^10^) rules and then to define the preference relation for them. Such a valuation would be almost completely arbitrary and computationally intractable. A further reason for rejecting this approach is that the aim here is to model the reasoning of the courts and the parties as faithfully as possible in relation to actual instances of argumentation and to provide explanations that are understandable by users. To that end, it is necessary to determine a certain “reasonable” subset of *P*(PROP). The present research questions are as follows:

- How can and should such a reasonable subset be determined?
- What should be the syntax of the rule's antecedents, and how is a relation of preference between these rules to be established?

In addressing these questions, the following general problem provides a context for our investigations. Let us assume that a researcher has succeeded in establishing an appropriate knowledge base for a given case (C), encompassing a reasonable set of defeasible rules, properly connected to valuations and related to each other by relation of preference. The present paper is concerned with the incremental building of such a generalised knowledge base, highlighting both the possibilities and the limitations of that development.

## 4. The Issue of Uniqueness versus Comparability of Cases from the Practical Perspectives of Judge and Mediator

This section discusses the possibility of analogizing from one case to another from the perspective of a lawyer or mediator. It is our opinion that these sources may prove useful in developing more fine-grained methods for CBR inferences in the interpretation of indeterminate concepts. In formulating a directive to treat each case as unique, the commonplace postulate concerning avoidance of routine in the work of a judge, lawyer, or mediator by determination of its factual and legal circumstances (known as diagnosis of the conflict) should be assumed to be methodologically justified. This conforms with the assumption that each human being is an individual, capable of forming unique impressions, desires, and relationships. Against earlier belief in the stable rationality of human motivations, judgments, and decisions, the present state of the art in psychology points to nonschematic patterns in the functioning of the human mind [34].

The influence of intuition, emotional style, heuristics, and social interaction on the thinking and behaviour of human beings means that parties to legal disputes are subject to many cooccurring internal and external determinants. These justify an individual approach to the investigation of any case, taking into account both legal and extralegal (i.e., psychological, communicational, relational, economic, social, and ethical) points of view [35, 36]. At the same time, the practice of law and mediation unambiguously shows that, in taking this approach, it is advisable to (1) compare different cases and disputes in their various aspects; (2) look for any common elements, similarities, or concurrence in the legal and factual features of these cases; (3) conduct comparative investigations concerning the motivations, needs, reasoning, and argumentation of agents in different cases, including those of intervening third persons (judges, arbiters, and mediators); and (4) categorise the different aspects of disputes. Such comparison fosters the optimisation and application of individually and socially appropriate solutions. A multicriteria analysis supporting diagnosis of the dispute enhances interpretation of the concept of the child's well-being in the given situation and any application of judicial and extrajudicial means in realising this value.

The analysed issue will seem obvious in democratic countries governed by law, given the existence of formally binding precedents and the* stare decisis* principle in common law systems, as well as the paradigm of equal treatment of citizens and the right to a fair trial in civil law systems. The comparison of cases is additionally strengthened by the principles of unity of interpretation and unified application of the law within both domestic legal systems and the system of law of the European Union [37]. Such a legal context reinforces work in the fields of ODR and AI and law research relating to amicable resolution of legal problems. It should be emphasised that this conclusion not only pertains to AI-enhanced systems but also is valid in relation to classical mediation. Fairness of outcome, understood as a solution generally accepted in similar situations, is perceived as an important value by parties to disputes.

In the practice of mediation, conducted without the obligatory evidentiary proceedings and qualification of the facts of the case under a valid norm (subsumption), the issue of similarity of cases gains a specific additional dimension. In respecting the voluntary and autonomous character of parties' decisions concerning the conduct of negotiations and the content of any decisions arising (including the agreement itself), the mediators (1) search for and compare common elements between the case in question and past cases (external analysis) and (2) categorise important aspects of both subject matter and agents in the present case (internal analysis). The results of this comparison and categorisation enable mediators to analyse the conflict and to propose solutions that have been tested against past cases and are therefore considered optimal. Categories enabling comparison of cases and the application of adequate, verified solutions include the following:

1. Conflicts and causes (e.g., conflicts of relations, of values, of factual investigations, of communication, or of interest) [38].
2. Framing of the dispute (e.g., as a matter of power, of rights, of interests and needs, of aspirations, of identity, or of conflict management) [39].
3. Conflict-handling modes or ways of dealing with conflict (e.g., competing, collaborating, compromising, avoiding, or accommodating) [40, 41].
4. Preferred styles of decision-making (e.g., thinking versus feeling) [42].
5. Personality types of the parties (e.g., on the Myers Briggs scale) [43].
6. Course and degree of escalation of the dispute.
7. Life, family, occupation, and social situation of the parties.
8. Levels and types of needs of the parties [44].
9. Psychological phase of the conflict (which is particularly important in family disputes involving divorce) [45].
10. Evidentiary and legal positions of the parties.

Comparative analysis of present and past cases through the prism of the above categories enables the mediator to adequately apply experiences and findings. For instance, appropriate identification and categorisation of the causes of conflict guide the choice of an optimal solution and the manner of the mediator's intervention [37]. The ventilation of emotions, method of negotiation, reality testing, and argumentation applicable to a given category can also be guided by approaches that have previously proved useful in achieving mutual concessions and settlements. In practice, it is also helpful to refer to agreements executed in similar cases, especially where these have been empirically verified and accepted by the courts. Analysis of similarities between cases enables the mediator to use previous positive and negative experiences to predict the likely behavior of the parties and the effectiveness of any chosen means of intervention. Additionally, professional development should emphasise the common practice of anonymization of information exchanged between negotiators, mediators, and lawyers concerning precedents or untypical cases, as well as the need to avoid error and to replicate tested and optimal solutions.

To conclude, while taking into account the individual and unrepeatable character of any human being and their situation, not every case need be considered essentially unique. Despite the diversity of human personalities, motivations, needs, and modes of thinking and argumentation and of the legal and factual features of cases, efficient comparison and categorisation are in practice possible and justified. In continental legal culture, such comparisons may at least provide fruitful examples for analysis of the case at hand [46]. The categories of comparison listed above can fruitfully be applied in developing the CBR module of PPSS.

## 5. Heuristics Used to Extract Preferences among Rules from Background Knowledge: General Remarks and Discussion of the Literature

Having discussed the methods employed by lawyers and mediators to compare cases and to categorise their important elements, we may now consider how these insights can fruitfully be employed in developing the PPSS knowledge base. In the existing version of PPSS [11], both propositional elements (options and environmental factors) and, in particular, defeasible rules (DR) and preferences between them are predefined within the system. The following defines a context for development of the automated generation of orderings of rules within the constraints of resemblance to actual legal argumentation (i.e., the generated rules should not be overly complex) and computational features of the system.

The intention here is to model the reasoning of a judge who must decide which case features should be counted as more decisive, leading to an assessment of the case with respect to the criterion of the well-being of the child. Such determinations inform continuous updating of the meaning and scope of the (indeterminate) concept of the child's well-being, although in civil law countries the impact of judicial decisions on future cases is much weaker than in common law jurisdictions, where past cases have the status of binding precedent. In the absence of such authoritative sources, both parties to the drafting of parenting plans and judges assessing those plans on the criterion of well-being of the child must use different heuristics to assess the relative importance of features of the analyzed cases. As different people may use different heuristics in addressing a given cognitive problem, it would be implausible to advance a set of universally used heuristics. The selection presented below is therefore to some extent arbitrary and clearly nonexhaustive. It was developed on the basis of the authors' experience of both theory and practice of legal cases involving family disputes within the framework of Polish law.

Most obviously, a heuristic may be defined as a nonalgorithmic problem-solving strategy (*locus classicus* [46]). In framing the PPSS knowledge base, we are less interested in the process of decision-making under uncertainty than in the process of grasping the important features of the analysed case and assessing their relative importance with regard to the criterion of well-being of the child. Given the defeasible character of reasoning based on heuristics, it is always possible that this may lead to a fallacious decision in concrete cases, accounting for the possible disagreement of PPSS users with suggestions and valuations provided by the system [47]. Our aim is to develop a knowledge base that will decrease the likelihood of such disagreements.

Some of the main heuristics used in determining preference relations between defeasible rules to interpret aspects of the concept of the child's well-being may be classified as follows.

*(1) Subjective Decision of the Agent Deciding on the Relation of Preference*. In such situations, establishing a relation of preference between rules is based on arbitrary or intuitive choices. In some instances, in the absence of any accessible source from which derive the preference relation and the grounds for establishing it, an arbitrary choice may be the only available option.

*(2) The Law (in the Broad Sense of the Term), Encompassing Statutory Sources, Case Law, and Doctrinal Elaborations*. In particular, judicial decisions are a rich source in establishing preference relations between rules for the interpretation of indeterminate legal concepts. However, in civil legal systems, the rules expressed in judicial decisions cannot generally be considered binding and cannot therefore strictly constrain the decision-making processes of interested parties or of the court. In these circumstances, relative preference relations between rules encompassing features of cases expressed in judicial decisions in civil law countries can be seen as no more than weak constraints on reasoning in new cases. Even striking similarities between new and old cases do not warrant application of the previously expressed preference relation, although they may in practice provide good reason for its application.

*(3) Commonsense Reasoning*. This provides an abundance of heuristics, applicable to a potentially infinite number of cases. In the first place, commonsense reasoning may play a negative role by eliminating absurd or unreasonable preference relations between rules and subsequently a positive role in seeking an ordering of rules that appears “most reasonable.” Clearly, these heuristics may lead to defeasible results.

*(4) Appeals to Morality and/or Teleological Reasoning*. This type of heuristic enables an agent to argue that rule *r*_1_ is preferred over rule *r*_2_, for example, because the former realises a more important value or because it realises some given value to a greater extent. Background knowledge related to values is a source of heuristics under the concept of balancing; generally speaking, a rule may be assessed as the preferred one where its application leads to the most favourable balancing of relevant values. The idea of balancing values naturally assumes that the realisation of one value may to some extent limit the possibility of fully realising other values. As the child's well-being should itself be treated as a value, we refer here to a set of lower-level values or goals that may contribute to the realisation of this paramount value (e.g., good communication between parents and children, good financial situation, or children's safety). In fact, these lower-level values are represented in particular dimensions of PPSS, although in a distributed manner.

There is an abundant literature (in AI, AI and law, and legal theory) concerning the establishment of preferences between arguments based on rules and comparison of sets of reasons in rule-based reasoning. For obvious reasons, it is not possible to discuss all these research streams in depth here. Instead, we will focus on selected relevant works to demonstrate how our approach contributes to the state of the art. It is important to emphasise that the problem of establishing preference relations between conflicting legal rules is beyond the scope of our investigations, which focus on relative preferences in the assessment of a given factual situation from the point of view of a single indeterminate legal criterion, in this case, the concept of well-being of the child.

In terms of general research on AI and argumentation, there exists a vast literature on preference-based argumentation. This work focuses on formal properties of developed theories, often within the frame of abstract argumentation. This idea has been introduced to a wider audience by Dung [48] and in theories concerning structured or instantiated argumentation. For instance, Modgil and Prakken [5] have recently presented a fully fledged account of argumentation with preferences, rooted in Dung's theory but capable of expressing a wide range of instantiations by means of the ASPIC^+^ formalism. Although this line of research is of great importance, its relevance for PPSS is limited at this stage of the system's development, as PPSS does not allow users to argue about relative preferences for rules by means of a computational model of argumentation. Rather, the aim of the PPSS inference engine is to infer the correct valuation of the given case and to present it to users of the system, with suggestions for changes to chosen options.

Another important precedent informing PPSS is the model of teleological reasoning incorporating cases and values developed by Bench-Capon and Sartor [19], which was designed to build theories of cases from background knowledge (encompassing,* inter alia*, cases and factors) and to evaluate those theories against a criterion of coherence. Although PPSS does not employ the notion of theory, an important aspect of that model for the PPSS knowledge base is the set of heuristics (referred to as “theory constructors”) used to infer certain theoretical elements from background knowledge. In particular, Bench-Capon and Sartor discuss procedures for the extraction of value preferences from cases, as well as rule preferences based on value preferences. In the present version of PPSS, there are no rules that would enable the system to abduct value preferences from the ordering of rules in cases. This is problematic because the formalistic and magisterial style of the Polish courts often makes it difficult to reconstruct the details of teleological reasoning. However, the existence of such considerations in certain judgments stored in the PPSS database opens the possibility of extending the inference engine in this way. In summary, it should be stressed that the heuristic character of establishing preference relations between rules in PPSS should be seen as (possibly justified) advice to users rather than as an authoritative solution.

## 6. Selected Technical Issues

It seems important to point out two disadvantages of the model presented in previous versions [11] of PPSS: the lack of discussion of possible utilisation of negation in the conditional part of defeasible rules and the necessity for manual extraction of defeasible rules' preferences. This section will address these problems, suggesting a first step towards automation of the process.

The problem of negation is of particular importance because, at the first sight, it is difficult to imagine a legal decision support system with no possibility of negation, given that negation appears so frequently in both legal and commonsense rules. Generally speaking, two kinds of negation may be distinguished in formal logic as follows:

- Classical (strong) negation (denoted by ¬) in the conditional part of the rule is fulfilled if it is known that a given proposition is not true.
- Negation as failure (denoted by ~) is fulfilled if every possible proof of a given proposition fails.

If we allow for utilisation of both kinds of negation in the conditional part of the rule, we may use a construction like ~ ¬  *P*, usually interpreted as “it is not known that not *P*.”

In [3], Sergot et al. describe a legal expert system that models the British Nationality Act, in which they use only negation as failure. The problem of double negations (including one classical negation) was solved by changing a negative literal (“X was not born in the UK”) into a positive one (“X was born outside the UK”). From another point of view, many other logical models of legal reasoning [21] allow for utilisation of both kinds of negation. Other expert systems (such as that used for evaluation of the quality of protection of IT systems [49]) allow for utilisation of negation as failure in the conditional part of the rule only and for classical negation in the consequence part of the rule.

It is important to understand which kind of negation (if any) is most suited for modelling defeasible rules in PPSS. Utilisation of negation as failure seems more flexible, and Sergot et al. [3] use it in their system. However, in the specific legal context of rules for evaluating the well-being of a child, where the chosen option has a positive character, the existence of environmental factors must be justified. Even the negated factor (e.g., “no cooperation between parents”) should be explicitly stated and strictly justified on the basis of evidence, making negation as failure unnecessary in this specific kind of legal rule. Moreover, as in [3], classical negation may be replaced by positive propositions or by propositions such as “no cooperation,” which may be treated in the same way as ordinary positive propositions. To bear on an assessment of options chosen by the negotiating parents, a lack of certain circumstances should be asserted as a “positive fact.” Such a conception of defeasible rules has interesting consequences, which will be discussed later.

It is trivial to say that a legal expert system incorporating an Explanation Module should accurately reflect the reasoning of a human lawyer. Defeasible rules in PPSS should also mirror rules by which a human actor evaluates the influence on one of the dimensions representing the well-being of the child of a chosen option and the context of a case as described by a set of factors. The same option in a different context may vary in its influence on a given dimension, making it important to choose the rule referring to the context at hand. This is important because the occurrence or nonoccurrence of one particular factor can critically alter evaluation of a given dimension.

**Example 1 .**

Let us consider two defeasible rules from the example presented in [11] as follows:

*r*_1_: *α*∧*δ*∧*γ*⇒3.
*r*_3_: *α*∧*δ*∧*γ*∧ef2⇒0.
*r*_1_, *r*_3_ ∈ DR(*Di*).

When *α*, *δ*, and *γ* are options, ef2 is environmental factor.

In our case, if we assume that factor ef2 exists and we choose all three options (*α*∧*δ*∧*γ*), the conditions of both rules will be satisfied, leading to a conflict. How can such a conflict be resolved? There are two possible ways; the first of these is to add the possibility of negation (negation as failure) of the condition of the rule, with additional conditions to rule *r*_1_, excluding the existence of ef2, as in Example 2.

**Example 2 .**

Consider

*r*_1_: *α*∧*δ*∧*γ*∧~ef2⇒3.
*r*_3_: *α*∧*δ*∧*γ*∧ef2⇒0.
*r*_1_, *r*_3_ ∈ DR(*Di*).

Such a solution has one important disadvantage; rule *r*_1_ (as well as *r*_3_) should also contain clauses excluding other factors that can alter evaluation of a chosen option. In real-life situations, there may be a number of such clauses, making such a rule counterintuitive and difficult to understand.

The second way to overcome the problem of conflicting rules is to assume that, in cases of conflict between two (or more) rules, one of them is preferred to the others, as in Example 3.

**Example 3 .**

Consider

*r*_1_: *α*∧*δ*∧*γ*⇒3.
*r*_3_: *α*∧*δ*∧*γ*∧ef2⇒0.
*r*_3_ > *r*_1_ ∈ ORD_C_.

There is a long history of ordering rules in formal models of legal reasoning. However, our case is specific. First, we must detect which rules are in conflict. Unlike advanced models of reasoning and argumentation, only one defeasible rule can be used in our model to evaluate the level of promotion of a given dimension. It follows that if a given case satisfies the conditions of more than one rule, then all these rules are in conflict. Second, we have to discover and justify the sources of order between conflicting rules. There may be many such roots, from legal principles like* lex specialis*,* lex superior*, or* lex posterior*; they may also come from preferences between values. These issues are extensively discussed in the literature, as, for instance, those in Sartor [50]. The case described in the above example is interesting because every case satisfying the conditions of rule *r*_3_ also satisfies the conditions of rule *r*_1_. On that basis, we may say that the conditional part of rule *r*_1_ subsumes the conditional part of rule *r*_3_. How are we to deal with such cases? The most intuitive way is to observe that rule *r*_3_ should be treated as an exception to the more general rule *r*_1_, and so, on the basis of the principle of* lex specialis derogat legi generali* (in which the specific act (provision) derogates from (prevails over) the general regulation [50]), we may assume that *r*_3_ is preferred to *r*_1_. Here, we are using this collision rule analogically, as the rules employed in PPSS will rarely have a strictly legal character; rather, they are “judicially authorised” commonsense rules, used by courts in issuing opinions. Of course, conflict may arise not only between rules with subsuming conditional parts; in such situations, preference relations between rules should be built on the basis of the other justifications. It is our opinion that conflict resolution by the ordering of rules seems more intuitive and better reflects human reasoning; for that reason, a generalisation and formalisation of this method are offered below.

On the basis of the above and [11], the following new elements can be added.

**Definition 4 (conflicting rules).**

If one has two rules *r*_*x*_ and *r*_*y*_  (*r*_*x*_ *r*_*y*_ ∈ DR(*Di*)) assigned to a Dimension *i*, and there exists a case that satisfies conditional parts of both of them, and the consequents of *r*_*x*_ and *r*_*y*_ are not identical, then rules *r*_*x*_ and *r*_*y*_ are in conflict.

On the basis of the above definition of conflict, we can create a conflict resolution method based on the analogical application of the collision rule of* lex specialis* as widely applied in the context of conflict between statutory rules. By applying this method, we can automatically add a new order to a set ORD_C_ that allows us to defeat conflicting and less preferred rules.

**Definition 5 (lex specialis).**

If one has two conflicting rules *r*_*x*_ and *r*_*y*_, and if

(i) *Ω* is a conditional part of the rule *r*_*x*_, which is a conjunction of options chosen by the parents (*Ω*_*X*1_) and circumstances of the case (conjunction of environmental factors *Ω*_*X*2_), and
(ii) the rule *r*_*y*_ has a conditional part of the form *Ω*∧*Ω*_*y*_, where *Ω*_*y*_ is a finite conjunctive formula encompassing elements taken from the set of EFs,

then rule *r*_*y*_ is preferred to rule *r*_*x*_, and the relation (*r*_*y*_ > *r*_*x*_) should be added to ORD_C_.

We may add that if *r*_*y*_ > *r*_*x*_, then the conclusion of *r*_*y*_ rather than the conclusion of *r*_*x*_ is counted as true in a given factual situation.

The above definitions require some additional comments. As the conditional part of defeasible rules is a conjunctive formula of positive propositions, creating a new rule by adding a new condition to an existing rule will narrow its scope. Such a new rule can be treated as a special exception to the old one; on the basis of the principle of* lex specialis*, then, it should take higher priority in cases of conflict than the old one. This is illustrated in Example 6.

**Example 6 .**

Let us assume the following rules from Example 1:

*r*_1_: *α*∧*δ*∧*γ*⇒3.
*r*_3_: *α*∧*δ*∧*γ*∧ef2⇒0.
*r*_1_, *r*_3_ ∈ DR(*Di*).
Set ORD_C_ is empty: ORD_C_ = *∅*.

Let us assume that, in our case, environmental factor ef2 occurs and that parties choose options: *α*, *δ*, and *γ*; hence, rules *r*_1_ and *r*_3_ are in conflict.

If we substitute *Ω* for the formula (*α*∧*δ*∧*γ*), then both our rules will take the following forms:

*r*_1_: *Ω*⇒3.
*r*_3_: *Ω*∧ef2⇒0.

As the conditions of application of* lex specialis* are satisfied, we add order *r*_3_ > *r*_2_ to set ORD_C_, thus resolving the conflict.

The model of* lex specialis* presented above enables avoidance of a special kind of conflict that may appear in the set of defeasible rules. Of course, it does not allow resolution of all possible conflicts that might appear in the DR set. If such a conflict appears, one of the other heuristics should be used to resolve it.

## 7. Conclusions

Despite impressive progress in the field of development of legal expert systems, it remains far from clear how such systems should be developed to make them useful in practice, in dealing with nontrivial cases. The present paper contributes to this line of research in a very specific context defined by the conjunction of the following elements: (1) continental legal culture, where there is no precedential constraint; (2) nonadversary proceedings, where parties are motivated to cooperate rather than to argue against each other but may still have diverging interests; and (3) highly indeterminate, as in the open-textured and evaluative concept of the well-being of the child.

Our main findings are as follows. It would be computationally intractable and descriptively inadequate to seek to apply exhaustive algorithms to interpret the well-being of the child by means of classical hybrid rule-based and case-based knowledge representation structures. To provide users with an explanation of outputs that simulates the reasoning of an actual lawyer or mediator, certain heuristics should be applied to provide defeasible rules interpreting this concept in concrete cases, as well as preferences between these rules. It is unnecessary to utilise any form of negation in the syntactical structure of these rules; the specificity of description of the case in the antecedent of the given rule provides a very convenient heuristic for establishing priorities between rules. As a matter of course, this proposal is subject to debate, not least because the specificity of rules utilised here falls within the domain of commonsense rather than of strictly legal rules. In particular, effective utilisation of the specificity principle requires the knowledge of engineering to structure the system of environmental factors to establish a hierarchy of more general concepts and exceptions thereof. The limitations of this approach will be the subject of future investigations relating to the PPSS project.
